# A Case of Bedside Ultrasound in COVID-19 to Prognosticate Functional Lung Recovery

**DOI:** 10.1155/2021/8847887

**Published:** 2021-01-23

**Authors:** Kathryn B. Bartlett, Lexis T. Laubach, Elizabeth M. Evans, Kevin R. Roth

**Affiliations:** Department of Emergency and Hospital Medicine/USF, Lehigh Valley Health Network, Morsani College of Medicine, Cedar Crest Boulevard & I-78, Allentown, 18103 PA, USA

## Abstract

*Introduction*. The fight against COVID-19 poses questions as to the clinical presentation, course, diagnosis, and treatment of the condition. This case study presents a patient infected with COVID-19 and suggests with additional research, that bedside ultrasound may be used to diagnose severity of disease and potentially, prognosticate functional lung recovery without using unnecessary resources and exposing additional healthcare professionals to infection. *Case Report*. A 46-year-old male presented to the emergency department (ED) with cough, fever, and shortness of breath. Chest X-ray showed patchy airspace opacities bilaterally. Rapid testing resulted positive for SARS-CoV-2. Bedside ultrasound showed abnormal lung parenchyma, with diffuse comet tail artifacts, consistent with interstitial pulmonary edema. Following a prolonged intubation, patient's abnormal lung ultrasound findings are resolved.

## 1. Introduction

The fight against COVID-19 poses many unanswered questions as to the clinical presentation, the clinical course, and the best way to diagnose, treat the condition, as well as prognosticate functional lung recovery. It has been previously suggested that COVID-19 is a continuum of disease with a variety of ultrasound lung findings that may suggest the seriousness of an individual's disease depending on where they fall on the continuum [1–4]. Bedside ultrasound may be a way to quickly and rapidly diagnose severity and progression of the disease without using additional resources and exposing additional healthcare professionals to the illness [5]. We will discuss a case of a 46-year-old male diagnosed with severe COVID-19 with complete recovery and return of normal lung ultrasound findings. This case study demonstrates that with additional research, bedside ultrasound could be a way to determine how the lung parenchyma has healed and the progression of recovery of the disease [2].

## 2. Case Presentation

A 46-year-old Caucasian male with a significant medical history of hypertension, hyperlipidemia, paroxysmal atrial fibrillation, and prior tobacco abuse presented to the emergency department (ED) with cough, fever up to 39.4°C (103 F), shortness of breath, and intermittent diarrhea. His symptoms began nine days prior to the presentation to the ED, with persistent fevers and dry cough and worsening shortness of breath. He was taking his home medications of benazepril, atorvastatin, and prn acetaminophen and had recently been placed on an albuterol MDI by his family physician without relief of shortness of breath.

**Figure 1:**
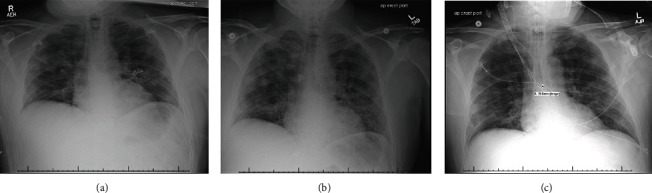
(a) Early chest X-ray (CXR) on day of admission consistent with patchy airspace infiltrates concerning for early viral pneumonia. (b) Two days into admission, CXR shows diffuse ground glass and interstitial opacities with worsening airspace infiltrates. (c) Decrease in diffuse airspace infiltrates and ground glass opacities, six days after intubation.

Upon arrival to the ED, the patient was noted to be febrile to 39.4°C (103°F), with tachypnea, respirations in the mid-20s, and SpO2 90-91% on room air. He was placed on two liters of oxygen via nasal cannula, and chest X-ray (CXR) showed patchy airspace opacities bilaterally, suspicious for viral pneumonia (Figure 1(a)).

The patient remained tachycardic intermittently into the 120 s. Laboratories were unremarkable, with the exception of potassium levels of 3.2 mmol/L, AST 118 U/L, ALT 101 U/L, and borderline hyponatremia of 132 mmol/L upon admission. Potassium was replaced, and intravenous fluid replacement corrected the hyponatremia. He started on levofloxacin, since his viral testing had not yet resulted. Within one day of admission, his oxygen requirements worsened from two to six liters of nasal cannula, with worsening tachypnea and work of breathing. Subsequent viral testing resulted positive for SARS-CoV-2, and levofloxacin was discontinued. He underwent testing of lactate dehydrogenase, D-dimer, ferritin, and C-reactive protein, all of which were elevated. Repeat CXR showed worsening patchy airspace disease and diffuse ground glass opacities two days after admission (Figure 1(b)).

The patient's hospital course was complicated by atrial fibrillation (AF), with rapid ventricular response (RVR). He started on metoprolol and eventually, as clinical condition worsened, required intravenous digoxin due to hypotension. Anticoagulation was started despite low CHADSVaSC-2 score, due to the possibility of emergent cardioversion if the patient became rapidly more hypotensive. Arterial blood gas measurements continued to show worsening hypoxemia, and the patient was then placed on high flow nasal cannula, with duonebulizers, as needed. Consistent with standards at the time, the patient was started on azithromycin and hydroxychloroquine and completed a 5-day course.

**Figure 2:**
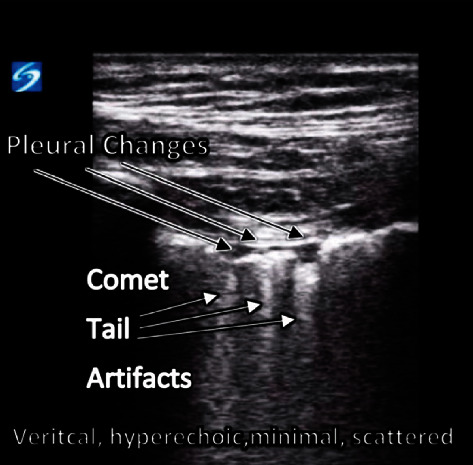
Confluent comet tail artifacts, as an example of interstitial edema, present in COVID-19 patients. This image is similar to the patient in this case study; however, it is not the same patient.

On day three of admission, the patient required intubation for persistent increased work of breathing and hypoxemia. Bedside ultrasound at this point showed abnormal lung parenchyma, with diffuse comet tail artifacts consistent with interstitial pulmonary edema (Figure 2).

**Figure 3:**
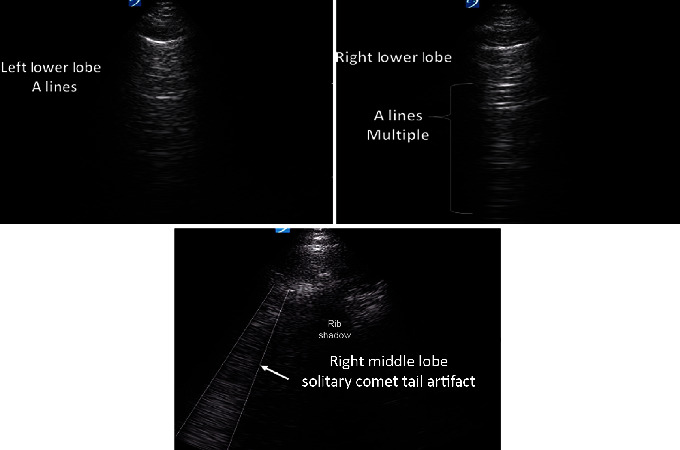
The patient's bedside ultrasound images after extubation revealed horizontal artifact consistent with normal lung parenchyma, and only a single comet tail artifact noted after bedside ultrasound of all lung fields.

Phenylephrine was started for persistent hypotension, as the patient could not tolerate other vasopressors due to AF with RVR. While intubated, he showed evidence of moderate acute respiratory distress syndrome, with a P/F ratio ~ 160 that ultimately worsened requiring paralytics and prone positioning. Liver enzymes peaked in the 200 s, with a negative hepatitis panel. Laboratories revealed a transient acute kidney injury, with a creatinine of 1.2 mg/dL from a baseline of 0.8 mg/dL. An echocardiogram revealed a normal ejection fraction, but a mildly enlarged right ventricular size, consistent with lung disease. On day three of intubation, the patient's P/F ratio began to improve, and he no longer required prone positioning. Paralytics were discontinued on day four of intubation. Six days after intubation, CXR showed improvement in the patchy diffuse ground glass opacities (Figure 1(c)). The patient underwent diuresis and was extubated to nasal cannula eight days after being intubated. However, he continued to intermittently self-prone due to persistent feelings of shortness of breath for several days after extubation. He was weaned off nasal cannula prior to discharge, and a bedside ultrasound shortly after extubation revealed only horizontal artifacts, with no or minimal comet tail artifacts, suggesting normalization of the lung parenchyma (Figure 3). The patient was discharged home with minimal home care needs four days after extubation.

## 3. Discussion

Patients are presenting with a wide range of clinical presentations with COVID-19 that seem to correspond to a continuum of abnormal ultrasound lung findings, such as confluent comet tail artifacts, pleural effusions, pleural wall thickening, and hepatization of lung parenchyma [1]. Standard imaging used to diagnose COVID-19 is CT vs X-ray, which requires that the patients be transported to the radiology department. This potentially exposes numerous other patients and healthcare workers in the process via airborne particles even with disinfection procedures [2, 6]. We have illustrated a case, in which a patient had patchy diffuse ground glass opacities noted on the CXR that initially worsened and then gradually improved throughout the hospital stay. When evaluated via bedside ultrasound after prolonged intubation, the patient's lung parenchyma had returned to normal. Future research to investigate if ultrasound could aid in diagnosis of severity of illness and, therefore, disposition, but also potentially prognosticate improvement [2, 5]. But certainly, this case study raises the question if ultrasound could be used to prognosticate improvement and functional lung recovery while mitigating the resources and those exposed during a pandemic.

## 4. Conclusion

Bedside ultrasound may be essential in sparing sparse resources and limiting exposure to the disease, while offering valuable immediate objective data. Future study is needed to fully understand the role that bedside ultrasound may play in diagnosis, disposition, and prognostication. This case illustrates the potential importance of further investigation regarding the role of bedside ultrasound for diagnosing severity of disease and prognosticating recovery in COVID-19 patients.
